# Improving peripheral venous cannula insertion in children: a mixed methods study to develop the DIVA key

**DOI:** 10.1186/s12913-022-07605-2

**Published:** 2022-02-17

**Authors:** Jessica A. Schults, Tricia M. Kleidon, Victoria Gibson, Robert S. Ware, Emily Monteagle, Rebecca Paterson, Karina Charles, Adam Keys, Craig A. McBride, Steven McTaggart, Benjamin Lawton, Fiona Macfarlane, Chloe Sells, Claire M. Rickard, Amanda J. Ullman

**Affiliations:** 1 The University of Queensland, School of Nursing, Midwifery and Social Work, Rm 318 Herston Campus, St Lucia, Queensland 4006 Australia; 2 Herston Infectious Diseases Institute (HEiDI), Metro North Hospital and Health Service, Brisbane, Australia; 3 Queensland Children’s Hospital, Children’s Health Queensland Hospital and Health Service, South Brisbane, Queensland Australia; 4 Alliance for Vascular Access Teaching and Research, Menzies Health Institute Queensland, Griffith University, Nathan, Queensland Australia; 5 School of Nursing and Midwifery, Griffith University, Brisbane, Australia; 6 Centre for Applied Health Economics, Griffith University, Nathan, Queensland Australia; 7 Department of Emergency Medicine, Queensland Children’s Hospital, South Brisbane, Queensland Australia; 8 Department of Emergency Medicine, Logan Hospital, Meadowbrook, Queensland Australia; 9 Royal Brisbane and Women’s Hospital, Brisbane, Queensland Australia

**Keywords:** Pediatrics, Catheterization, peripheral, Difficult intravenous cannula insertion, Clinical decision-making, Decision support techniques, Quality improvement

## Abstract

**Objective:**

To develop and validate a difficult intravenous access risk assessment and escalation pathway, to increase first time intravenous insertion success in paediatrics.

**Methods:**

Mixed methods underpinned by literature and co-production principles. Iterative development of the instrument was informed through semi-structured interviews and stakeholder workshops. The instrument includes a risk assessment, inserter skill self-assessment, and escalation pathways. Reproducibility, reliability, and acceptability were evaluated in a prospective cohort study at a quaternary paediatric hospital in Australia.

**Results:**

Interview data (three parents, nine clinicians) uncovered two themes: i) Recognition of children with DIVA and subsequent escalation is ad hoc and problematic; and ii) Resources and training impact inserter confidence and ability. Three workshops were delivered at monthly intervals (February–April 2020) involving 21 stakeholders culminating in the co-production of the “DIVA Key”. The DIVA Key was evaluated between May–December 2020 in 78 children; 156 clinicians. Seventy-eight paired assessments were undertaken with substantial agreement (concordance range = 81.5 to 83.0%) between the assessors. Interrater reliability of the DIVA risk assessment was moderate (kappa = 0.71, 95% CI 0.63–0.80). The DIVA Key predicted multiple insertion attempts for red (high risk) DIVA classification (relative risk ratio 5.7, 95% CI 1.2–27.1; reference low risk). Consumer and clinician satisfaction with DIVA Key was high (median (IQR) = 10 [8–10]; 8 [8–10 respectively).

**Conclusion:**

The DIVA Key is a straightforward, reliable instrument with inbuilt escalation pathway to support the identification of children with difficult intravenous access.

**Supplementary Information:**

The online version contains supplementary material available at 10.1186/s12913-022-07605-2.

## Introduction

Insertion of a peripheral intravenous catheter (PIVC) is almost synonymous with hospitalisation [1, 2]. As a vascular access device, it is minimally invasive and facilitates immediate medical treatment. However, most children and their families describe insertion of a PIVC to be one of the most painful and stressful procedures during their hospitalisation [3]. Up to 69% of first attempt insertions fail [4–6], leading to delays to medical treatment and extended inpatient days [7, 8]. For health services, PIVC insertion failure contributes to significant usage and wastage of healthcare resources, costing the Australian health care system nearly $450 million [AUD] annually [9].

More than 50% of children are conservatively estimated to have difficult intravenous access (DIVA) [5, 10, 11]. DIVA is characterised by nonvisible and non-palpable veins; which may be due to physiology, pathology, or previous PIVC damage; making PIVC insertion ‘difficult’ for most clinicians [6]. The ‘average’ PIVC insertion requires two attempts and 20–30 min [12]. For children with DIVA, successful PIVC insertion may require upwards of nine attempts (needle sticks) [5]. Historically, DIVA status is retrospectively assigned after the patient has endured multiple failed PIVC insertion attempts. As a result, there has been a recent surge in the development of DIVA decision-making resources for paediatric patients [13]. Processes to identify children with DIVA have been attempted, primarily within emergency departments [14] with the development of three [11], four [10, 11, 15] and five variable *DIVA Scores* [16]; a *Peripheral Venous Grading System* [17] and a *Peripheral Vein Assessment Instrument* [18]. While many have been based on sizeable cohorts, their clinical generalisability in general hospital wards can be limited [10, 11, 15]. Furthermore, they are i) limited in their capacity to direct clinicians on how to manage these ‘DIVA’ patients once identified; and ii) typically do not feature consumer engagement, or patient centred principles such as procedural pain, or skill and confidence of the inserter. Together with input from key clinical stakeholders and pediatric consumers, we sought to develop a DIVA identification and escalation instrument (the DIVA Key) to support clinical practice. The reliability, reproducibility, and acceptability of the DIVA Key, as a strategy to identify children with DIVA, was then evaluated to assess clinical utility and application feasibility in paediatric settings.

## Methods

A sequential, mixed methods study was undertaken at the Queensland Children’s Hospital (QCH) Australia, a quaternary paediatric hospital, between February and December 2020. Study design was underpinned by the Co-production and Prototyping framework for Public Health Interventions [19]. Ethical approval was obtained from Children’s Health Queensland Human Research Ethics Committee (LNR/19/QCHQ/55326) and Griffith University (2019/797). The study is reported in line with The Strengthening the Reporting of Observational Studies in Epidemiology (STROBE) Statement [20] and informed by the COnsensus-based Standards for the selection of health status Measurement INstruments (COSMIN) checklist [21].

### Phase 1: *DIVA key* co-production

#### Aim

To co-produce an instrument to identify and escalate insertion procedures for children with DIVA.

### Research questions

1. What are the current experiences of key stakeholders regarding DIVA?
2. What are the instrument requirements based on stakeholders’ views?

#### Stakeholder mapping and recruitment

We identified and invited a cross-section of multidisciplinary stakeholders, including physicians, vascular access specialists, nurses, educators, quality and safety experts, hospital executives and consumer representatives to attend interviews and advisory group workshops via email invitation and expressions of interests (facilitated through QCH Family Advisory Council). Relevant multidisciplinary stakeholders were clinicians actively involved in vascular access with an interest in the development of policy and instruments related to identifying patients with DIVA across the health service. Where specific stakeholders were unable to attend, we employed snowballing techniques to recruit other relevant stakeholders. This approach provided a broad skill-set and perspective in the process of co-producing the instrument.

#### Semi-structured interviews

Semi-structured interviews were conducted to understand stakeholders’ current views and previous experiences with children with DIVA and related policy. Interviews utilised an interview guide and were conducted until data saturation was achieved, determined through the use of field notes [22, 23]. This activity facilitated evidence gathering of the current circumstances to be used as testimonials during the workshops [24].

Qualitative data obtained from interviews were analysed using iterative and inductive thematic analysis [23], per Braun and Clarke’s six phases of thematic analysis [23]. Initially two researchers (KC, JS) read transcribed interviews, independently generating initial codes. An audit trail was used to enhance dependability [25]. Codes were collated into potential themes. Themes were reviewed by both researchers in relation to coded extracts and a thematic map generated. To ensure authenticity resulting themes were reviewed by a third team member (VG).

#### Workshop design

Workshop design (*n* = 3) was informed by Carney/Oliver co-production principles [24, 26, 27]. Workshops were carefully planned using a scripted approach (example Supplementary material 1) and focused on joint decision-making between the research team, stakeholders, and consumers [28]. Workshop participants were provided an evidence summary (literature review findings [13]), excerpts from interview texts summarising desired instrument requirements, and an overview of current local DIVA policy. Expert opinion was an important consideration for co-production of instrument inclusions, with clinician gestalt linked with DIVA status prediction and first attempt insertion failure [29].

### Evaluation of instrument reproducibility, reliability, utility and acceptability

Reproducibility, reliability, utility, and acceptability of the instrument was evaluated using a prospective cohort study in the medical and surgical wards and operating theatres at QCH with the following objectives:

1. To evaluate the degree of agreement between inserters using the paediatric DIVA instrument (peripheral vein assessment instrument);
2. To evaluate the validity reliability of the paediatric DIVA instrument (peripheral vein assessment instrument);
3. To evaluate the reliability of the paediatric DIVA instrument (peripheral vein assessment instrument);
4. Describe the utility of the DIVA escalation pathway;
5. Determine clinician and consumer acceptability of the DIVA instrument;
6. To determine the performance of the DIVA instrument.

Staff resourcing and existing model of care prevented testing of the reliability and impact of the escalation component of the DIVA Key.

#### Sample and participants

A stratified, purposeful sample [30] of 78 children were recruited across the ages of: neonates (≤1 month) (*n* = 10), 1 month–2 years (*n* = 17), 2–5 years, 5–10 years (n = 17), and 10–18 years (n = 17). Children were eligible for study inclusion if they required the insertion of a PIVC. We excluded children requiring an emergent PIVC insertion, PIVC insertions outside hospital settings, children under the care of the Department of Social Services, and children from non-English speaking families without access to an interpreter.

#### Measurements

Study measures are outlined in Table 1.

**Table 1:** Measures and timeframes

Construct	Measure	Source	Time point			
			0	1	2	3
*Face validity*(33)	Five clinicians^b^ rated how well the instrument appeared to support PIVC insertion and DIVA recognition using a 5-point Likert scale 1 (strongly disagree) - 5 (strongly agree).	Cl	^a^			
*Content validity* [31, 32]	Five experts examined the DIVA Key’s content validity using measures of relevancy, clarity, and simplicity for each item.	Cl	^a^			
*Agreement* [33]	Interrater agreement of the instrument was assessed using percentage concordance (agreement parameter) between the assessors.	CRF		^a^		
*Reliability* [34]	Instrument reliability of the DIVA Key was assessed using Kappa.	CRF		^a^		
*Consumer acceptability*	Consumers (patient if > 8 years and/or parent representative) will be asked to rate their satisfaction with the DIVA instrument and escalation pathway (2 measures) using an 11-point numerical scale (0–10).	P, C			^a^	
*Clinician acceptability*	The inserter (clinician) will be asked to rate their satisfaction with the DIVA Key (peripheral vein assessment instrument and escalation pathway) using an 11-point numerical scale (0–10) and filed notes.	Cl			^a^	
*Utility*	PIVC insertions that are referred to an advanced practitioner that go on to be inserted by an advanced practitioner.	CRF			^a^	
	PIVC insertions requiring USG that receive USG technology	CRF			^a^	
*Performance*	First time insertion success: The number of PIVCs successfully inserted on first needle puncture as evidenced by blood flashback and ability to infuse 2-10 mL (age appropriate) 0.9% sodium chloride without signs of swelling or pain at the insertion site [18, 35, 36].	CRF			^a^	
	Total number of PIVC insertion attempts (skin punctures) to successfully insert PIVC [36].	CRF			^a^	
	PIVC failure prior to the completion of therapy, per 1000 catheter days [37].	iEMR				^a^

*CRF* Case report form, *iEMR* Integrated electronic medical records, *P* Parent reported measure, *C* Child reported measure, *Cl* Clinician, *Time point 0* Instrument development, *Time point 1* At point of identification of indication for *PIVC*, Time point 2 Post PIVC insertion; Time point 3 PIVC removal or failure. *DIVA* Difficult intravenous access, *PIVC* Peripheral intravenous catheter; *USG* Ultrasound guidance

^a^Measure administered at this time point

^b^Two doctors, three nurses

#### Study procedures

Patients who met eligibility criteria were approached for informed consent by the clinical research nurse (CRN). Peripheral vein assessments were then consecutively performed by two clinicians experienced in paediatric PIVC insertion. The order of the assessments was random, and successive, with assessors masked to the outcome of the previous assessment. Each patient’s ‘risk’ level on the vein assessment instrument was then referenced against the escalation pathway and the decision to comply with its recommendation was based on the inserter’s preference. Following PIVC insertion, the CRN assessed staff and consumer satisfaction with the instrument and overall PIVC insertion.

#### Sample size

We assumed the true concordance rate was 75%, and therefore to estimate the percentage concordance to within +/− 10%, with alpha = 0.05, a sample of 76 participants was needed. To ensure equal split among groups, however, the target sample size was set at 78.

#### Data collection and management

A screening log recorded patient information including name, unique hospital identifier (UR), eligibility and recruitment. Demographic and clinical data including age, gender, diagnosis, instrument assessment and recommendation were recorded on the case report form by the CRN and entered into an electronic data platform, REDCap™ (Research Electronic Data Capture) [38, 39].

### Statistical analysis

Patient and clinical variables, and staff and consumer satisfaction ratings, were summarised using descriptive statistics. Mean and standard deviation were used for normally distributed data, and median and interquartile range for data not normally distributed [31]. Counts and percentages were used to summarise instrument and pathway utility and feasibility. A mixed-effect logistic regression was used to analyse how often different clinicians reach the same response for each pair (insert or refer). Percentage concordance is a standard measure of the predictive accuracy in a logistic regression model. Reliability of the escalation pathway was analysed using kappa coefficients and 95% confidence intervals. Predictability of first insertion success by DIVA status was analysed using a multinomial logistic regression model.

## Results

### Phase 1: co-production of DIVA key

#### Semi structured interviews, gathering evidence of current practice

In total 12 stakeholders (three consumer representatives and nine clinicians) participated in semi-structured interviews. Analysis of interview data revealed two main themes which described the current clinical landscape: i) Recognition of children with DIVA and subsequent escalation is ad hoc and problematic; and ii) Resources and training impact inserter confidence and ability. Supplementary material 2 outlines the thematic map, supportive evidence and key principles and recommendations. These recommendations were used in conjunction with the literature review findings to support the co-production workshops.

#### Co-production of the DIVA key

Three workshops were delivered at monthly intervals (February 2020 to April 2020). Overall, 21 stakeholders participated in the workshops, of which nine attended two or more workshops. Stakeholders represented local and regional perspectives across Queensland Health, Academia and Educational organisations. By workshop two, stakeholders had co-produced a prototype of a paediatric DIVA vein assessment instrument and escalation pathway. Instruments were refined and finalised for testing in workshop three. Informal feedback received post workshops revealed stakeholders perceived their contribution as valuable to ensure the components of the instrument were user-friendly and relevant to the health service,

‘*It was great to be able to contribute my knowledge of procedural anxiety to the instruments development.’* (M3), and.

‘*The traffic light system was a good addition to the instrument layout and I was impressed they took my suggestions onboard and included it in the final instrument.’* (M9).

**Fig. 1:**
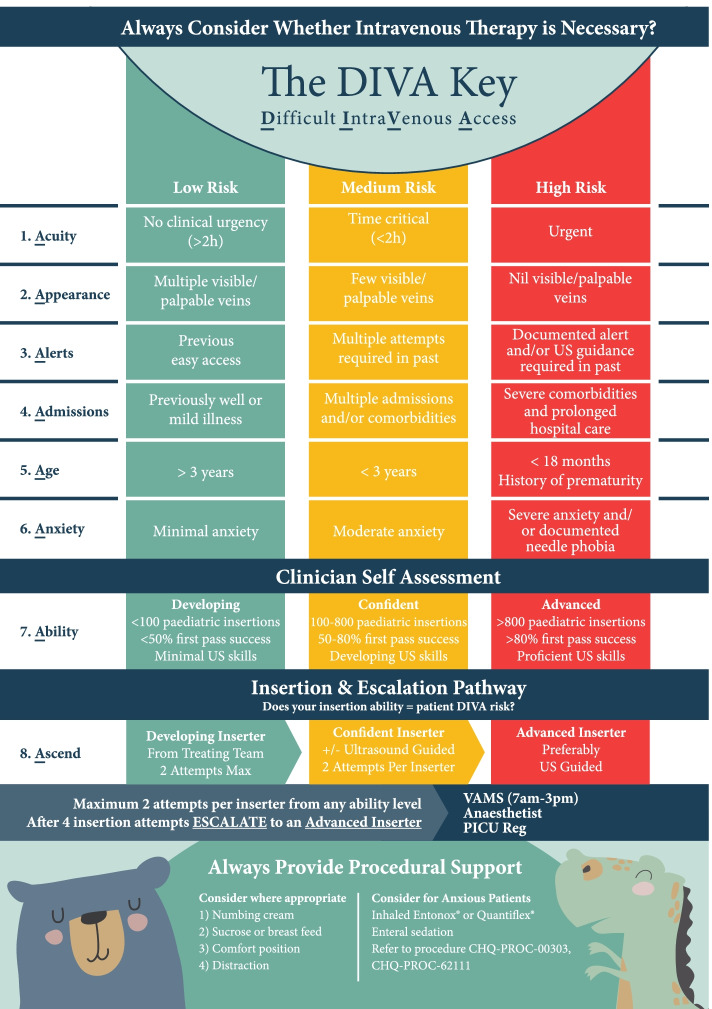
DIVA Key

The resulting instrument, entitled the **DIVA Key** is presented in Fig. 1 (visual concept created by @doubler.design). The instrument includes: i) DIVA risk assessment based on known DIVA risk factors [4, 15, 32, 33]; ii) inserter self-assessment of skill [34]; and iii) an escalation pathway [18]. A colour coded, traffic light system relative to risk (e.g., high risk of DIVA = Red) was used in addition to paediatric friendly graphics and logical flow to support decision making. Additional prompts for pain management and anxiety are included [35]. An outcome of the co-production process was that the instrument was tailored to the local health setting by including local policy references and contact details.

- Insert Fig. 1. DIVA Key

### Phase 2: evaluation of the DIVA key

#### Validity

Face validity of the instrument was demonstrated with a median rating of 4.5 (IQR 3–5) for clarity and relevance across five assessors (multidisciplinary clinicians who did not participate in the co-production workshops). The content validity of the DIVA Key resulted in all items scoring > 3 for clarity and feasibility using a 4-point level of agreement (1, not; 2, somewhat; 3, quite; 4, highly) [36, 37]. Revisions to flow, item wording, and the addition of more elements were proposed (e.g., traffic light system and procedural pain advice). The DIVA Key was subsequently amended to support the proposed modifications. Item validity was determined using a content validity index (I-CVI) [36, 37]. A panel of experts (*n* = 4) comprising multidisciplinary paediatric vascular access specialists were asked to provide feedback on the appropriateness and relevance of survey items using a four-point level of agreement (1, not; 2, somewhat; 3, quite; 4, highly). I-CVIs were calculated as the number of experts giving a score of 3 or 4 (item cut-off score of 0.75). Briefly, I-CVI for the DIVA Key ranged from 0.75 to 1.0. Overall, 20 out of 24 items in the DIVA Key had an I-CVI of 1.00, demonstrating moderate agreement among the content experts.

### Reproducibility, reliability, utility and acceptability

In the prospective cohort study, 78 children were recruited between May and December 2020 with no refusals or loss to follow up. The sample characteristics are outlined in Table 2.

**Table 2:** Sample characteristics

Variable	Participants (***n*** = 78)
Age, median (IQR), years	2.5 (0.4–9.0)
Male, n (%)	45 (58%)
Weight, median (IQR), kg	14.0 (7.0–31.0)
General appearance, adiposity, n (%)	
Minimal	28 (36%)
Moderate	33 (42%)
Excessive	17 (22%)
History of prematurity, n (%)	
No	70 (90%)
Yes	8 (10%)
Primary diagnosis, n (%)	
Medical	60 (77%)
Surgical	15 (19%)
Other	3 (4%)
Location of insertion, n (%)	
Medical ward	39 (50%)
Operating room	15 (19%)
Babies ward	10 (13%)
Surgical ward	8 (10%)
Other^a^	6 (8%)
Device inserted for, n (%)	
Treatment (e.g., antibiotics)	47 (60%)
Replacement device	17 (22%)
Other	14 (18%)

*IQR* Interquartile Range, *kg* Kilograms; *Mons* months, *Yrs* Years

^a^includes intensive care admissions

#### Agreement and reliability

Agreement of the 78 paired DIVA Key assessments was undertaken (individual assessors were blinded to the other assessment) with substantial concordance (range 81.5 to 83.0%) between assessors across low, medium, and high-risk groups (Table 3). Interrater reliability of the DIVA risk assessment (DIVA classification) was 0.71 (Kappa, 95% CI 0.63–0.80; p = < 0.001) suggesting moderate agreement. Interrater reliability of the DIVA Key recommendation for management was moderate 0.65 (0.57 to 0.72).

**Table 3:** Concordance of overall DIVA risk assessment by Clinician 1 and Clinician 2

First Assessor	Second Assessor			
	Low-risk	Medium- risk	High-risk	Specific Agreement (*of DIVA risk assessment*)
Low-risk	22	6	0	83.0% (72.1 to 93.9%)
Medium-risk	3	31	5	81.6% (72.1 to 91.1%)
High-risk	0	0	11	81.5% (65.5 to 97.4%)

When the DIVA Key identified a child as being medium risk, then the relative risk ratio (RRR) of having multiple insertion attempts, compared to if they were identified as low risk, increased by 6.2 (95% CI 1.6–24.5; *p* = 0.009). If the child was identified as high risk, compared to low risk, then the relative risk of having multiple insertion attempts was increased by a similar magnitude (RRR 5.7, 95% CI 1.2–27.1; *p* = 0.03).

### Acceptability

Consumer and clinician satisfaction with the DIVA Key was high. The median consumer satisfaction score was 10/10 (IQR 8–10). Clinicians reported a median satisfaction score of 8/10 (IQR 8–10) noting ‘*People might over-rate confidence.’* and ‘*More information needed around who makes the first attempt.’*.

### Instrument performance and utility

PIVC insertion characteristics and outcomes, by DIVA risk are outlined in Table 4. First attempt insertion success (46%) was lowest in children assessed as high risk (red) and highest in children classified as low risk (82%). The median number of insertion attempts in the high risk group was 2 (IQR 1–5) compared to 1 in the low and medium risk groups (IQRs 1–1 and 1–3 respectively). Insertion difficulty was rated highest in children classified as high risk (5/10, IQR 3–7). All children (*n* = 11) identified as high risk of DIVA had their PIVC successfully inserted by an advanced inserter (self-capability assessment RED). Of the 11 children, 90% (*n* = 10) of insertions utilised ultrasound guidance. No PIVC insertions were abandoned with all children receiving an intravenous catheter for treatment.

**Table 4:** Comparison of PIVC insertion characteristics and outcome, by DIVA risk (assessed by treating clinician)

Variable	Low (green) (***n*** = 28)	Medium (Yellow) (***n*** = 39)	High (Red) (n = 11)
First attempt insertion success, n (%, 95 CI)	23 (82%; 68–96)	23 (59%; 46–74)	5 (46%; 16–75)
Number of attempts, median (IQR)	1 (1–1)	1 (1–3)	2 (1–5)
First attempt by^a^, n (%)			
Developing inserter	6 (21)	3 (8)	0 (0)
Confident Inserter	15 (54)	15 (38)	4 (36)
Advanced inserter	7 (25)	21 (54)	7 (64)
Successful PIVC placed by, n (%)			
Developing inserter	6 (21)	2 (5)	0 (0)
Confident Inserter	16 (54)	14 (36)	0 (0)
Advanced inserter	7 (25)	23 (59)	11 (100)
Rating of insertion difficulty^b^, median (IQR)	2 (1–3)	2 (1–5)^c^	5 (3–7)

*IQR* Interquartile range

^a^Self-report assessment of skill; ^b^ Successful inserter scale of 1–10; ^c^ 3 missing; ^d^day cases recruitment common

## Discussion

This project co-produced and validated the DIVA Key instrument to support the assessment and subsequent escalation of PIVC insertion care in children with DIVA. Consistent with the evidence-base around DIVA risk factors and existing DIVA instruments [10, 11, 13–18], the DIVA Key included objective descriptions of vessel quality (e.g., appearance) and practice variables (e.g., acuity, previous access history). These indicators were uniquely complemented by rating the child’s reported or perceived anxiety, along with the clinician’s self-assessment of ability, and recommendations for management of procedural pain and anxiety. In this study the DIVA Key demonstrated interrater reliability and agreement and was acceptable to both clinicians and families. Our findings support the instrument’s application and utility across a quaternary paediatric hospital setting.

Our prior literature review and survey of practice [13, 40] demonstrated a variability surrounding the sensitivity and useability of paediatric DIVA instruments. Whilst most tools demonstrated moderate predictive ability (DIVA Score area under the curve [AUC] 0.67 75% [15], 3-variable DIVA score AUC 0.72 [11]), clinometric testing outside of the emergency department was scarce. Further existing DIVA tools lacked decision-making cues to direct escalation, failed to consider inserter skill and confidence, and did not take into consider patient experience or preference (i.e., patient reported pain and anxiety). To overcome these obstacles, the DIVA Key was co-produced with stakeholders and consumers, and underpinned by the diverse and unique insights they provided [41]. The resulting resource, the DIVA Key, reflects the experiences of clinicians and consumers, and demonstrates promising reproducibility, utility, and acceptability. Due to the insights from stakeholders and consumers, the DIVA Key had strong face and content validity, and stakeholders qualitatively reported that they felt their feedback was incorporated into the final instrument.

PIVC insertion in patients with DIVA is challenging, even for experienced clinicians [42]. Irrespective of inserter skill, identification of DIVA risk should occur prior to subjecting a child to multiple, painful failed insertion attempts [43]. Early identification of children with DIVA relies on instruments that, even with minimal training, provide an objective and reproducible description of a child’s risk of DIVA. To achieve this, the DIVA Key employed alliteration ( [44] drawing emphasis to certain ideas) to prompt recall by users of DIVA risk factors (acuity, appearance, alerts, admissions, age, and anxiety) and a traffic-light system [45] for risk level, complemented by the clinician self-assessment (ability). As a result, there was considerable agreement among assessors of varied skill level when assessing the child’s predicted risk of DIVA, and a high level of inserter-reported satisfaction with the instrument. Additionally, the DIVA Key demonstrated high construct validity. Children who were assessed as ‘high risk’ had the lowest rate of first-attempt insertion success, slightly higher median insertion attempts, and greater perceived insertion difficulty.

Early identification of DIVA in children alone is not sufficient to improve patient outcomes. Clinicians in this study voiced concerns that they lacked the skills, training, or resources to manage patients with DIVA. This finding is consistent with earlier research highlighting the lack of support or resources available to clinicians once DIVA is identified [13]. Therefore, the DIVA Key includes a clear and concise escalation pathway (ascend), that matches patient level of risk and inserter competency, to guide the number of attempts before escalation (i.e., no more than 2 attempts prior to escalating to a more experienced clinician with or without vessel visualisation technologies). Similar to Hallam’s vessel preservation tool [18, 46].

This study has several strengths. Instrument development was grounded in a literature review, survey of practice and extensive stakeholder consultation [47, 48]. Additionally, the instrument development was underpinned by the co-production framework [19] and develop iteratively which allowed for constant adaption and improvement based on key stakeholder requirements. Despite these strengths our study is not without limitations. The development of the DIVA instrument relied heavily on clinical expertise. Although this level of evidence can be inconsistent, it was necessary to ensure the utility and acceptability of the instrument. Previous research demonstrated the complementary role of clinical ‘gestalt’ in accurate DIVA identification [29]. Finally, in this study, inserters were typically resident medical officers or registered nurses as is standard in our local setting. Insertion practices vary between institutions globally however, and interpretations of the concordance and utility of the instrument should be interpreted accordingly. The design of the current study precluded evaluation of the criterion validity of the DIVA Key, and therefore we were unable to determine the sensitivity and specificity, and corresponding positive and negative predictive values, of the DIVA Key in detecting a child’s DIVA status prior to escalation. Given the indicators of DIVA used in the DIVA Key are consistent with other highly sensitive paediatric instruments (e.g., Cornell Assessment of Pediatric Delirium [49, 50]), and the results of logistic regression found that first attempt insertion success was lowest in children assessed as high risk of DIVA, however, suggests that the DIVA Key is a promising instrument for the accurate identification of DIVA in this population.

## Conclusion

We co-produced a user-friendly, consumer focussed instrument to support the identification of DIVA in children, with an inbuilt inserter escalation pathway. In this cohort the DIVA Key appears to be a reliable instrument to support the identification and management of DIVA in children. Further testing among varying cohorts is warranted to further test generalisability of this measurement instrument and determine whether the implementation of an escalation pathway improves patient outcomes.

## Supplementary Information

**Additional file 1.**

**Additional file 2.**

## Data Availability

The datasets generated and/or analysed during the current study are not publicly available due local governance regulations and patient confidentiality but are available from the corresponding author on reasonable request.
