# Supplementary material for: Improving peripheral venous cannula insertion in children: a mixed methods study to develop the DIVA key
Source: BMC Health Serv Res. 2022 Feb 17;22:220. doi: 10.1186/s12913-022-07605-2 (PMC8851754; doi:10.1186/s12913-022-07605-2)
Supplement: Supplementary file 1 — Additional file 1. [file 12913_2022_7605_MOESM1_ESM.pdf]

Supplementary material 1. DIVA workshop aims and example workshop script

Workshop aims:

1. Build a working relationship between stakeholders and the research team; current practice and limitations; generate rich and valuable interaction to inform development of DIVA instrument;
2. Develop instrument inclusions (e.g., risk factors) and functionality (e.g., flow and colour grading) of the DIVA instrument; clear specification on outputs in terms of vein assessment and escalation pathway;
3. Focus on the design and co-production of a realistic DIVA instrument (previously adapted by stakeholders); explore instrument usefulness and refine inclusions and presentation.

EXAMPLE WORKSHOP 1. *Purpose: to identify stakeholders' expectations of the DIVA instrument; to identify what is working well and not so well; future hopes for the instrument; to review international examples of DIVA instruments and recognise risk factors for DIVA.*

Time: 2 hours
Location: Microsoft Teams® Meeting
Materials:

1. PowerPoint presentation of interview feedback, scoping review findings of international instruments, current healthcare policies related to PIVC insertion;
2. Paper and pen for note taking

Outputs:
List of stakeholders' comments regarding what is working well and not so well. Summary of discussion of the strengths and limitations of existing hospital policy for adaption
Roles:

1. Facilitator with knowledge of the topic
2. Scribe to document the session

Steps:

1. Participants introduce themselves and articulate their interest in DIVA. Facilitator explains and introduces the workshop content.
2. Facilitator provides an overview of the interview findings and scoping review of international diva instruments.
3. Stakeholders have 15 minutes to discuss these findings, strengths and limitations of instruments identified in the literature and the necessary inclusions of a future diva vein assessment and escalation instrument.
4. Facilitator attempts to identify themes and summarises discussion.
3. Facilitator presents proposed instrument for adaption: The peripheral vein assessment instrument developed by the Vessel Health Preservation (VHP) Group (31) used with permission from Hallam.
5. Stakeholders discuss for 30 minutes to identify strengths, weaknesses, and areas for adaption.
6. Facilitator summarises discussion and outlines plan for next workshop.
7. Scribe records or comments.

Opportunity for stakeholders to provide additional feedback through email follow-up, after the workshops.

DIVA: Difficult intravenous access
