# Supplementary material for: Improving peripheral venous cannula insertion in children: a mixed methods study to develop the DIVA key
Source: BMC Health Serv Res. 2022 Feb 17;22:220. doi: 10.1186/s12913-022-07605-2 (PMC8851754; doi:10.1186/s12913-022-07605-2)
Supplement: Supplementary file 2 — Additional file 2. [file 12913_2022_7605_MOESM2_ESM.pdf]

Supplementary material 2. Thematic map and key principles

Key theme	Subtheme	Supportive evidence
**Recognition of children with DIVA and subsequent escalation is *ad hoc* and problematic**	Recognition often occurs after the first failed attempt	Clinicians reported encountering children with DIVA frequently; up to '*25 – 75% of patients.'* (C2, C5), dependent on patient population.<br>The *'just have a go'* attitude persists despite increasing awareness children with difficult intravenous access require more skilled inserters
	Identification of and responsibility for escalation is a hospital wide issue	Clinicians discussed a lack of support to identify and manage DIVA. You '*have to be self-sufficient on the ward'* (C7), and '*It's difficult for us as Fellows, because there is no established escalation pathway.*' (C5).
	Consumer attitudes and experience of difficult PIVC insertion remain unchanged	Parents described presenting to the hospital for medical treatment for 'years' yet continually experiencing multiple PIVC insertion attempts, '*They were getting the people coming in and trying a few times and then having to go out.*' (P2), as stressful: '*He gets very stressed out during this process, which, in turn, if he's sick, can trigger other medical episodes, such as adrenal crisis.*' (P1). Parents also described escalation as problematic: there's '*…no escalating… they manage it very poorly*.' (P3).<br>Consumers indicated their anticipation at being able to contribute to the development of new, supportive policies for children with DIVA '*To be included in creating a benefiting tool for children with DIVA would be valuable'* (Pa 1)

Key principles and recommendations derived from interview data to inform instrument development

1. Enable the expanded use of DIVA policies to support not only the recognition of children with DIVA but also consistent escalation to an appropriate and skilled inserter, with or without ultrasound capabilities.

Recommendation: The DIVA instrument needs to include both i) DIVA identification based on existing risk factors, and ii) escalation pathway to ensure appropriate referral and inserter skill level.

<table>
<tr><td colspan="3">2. Consumers are ‘flying blind’ in the healthcare system with regards to difficult intravenous access and should be/want to be more involved in the decision making around PIVC insertion
Recommendation: Include consumers in the development of the DIVA instrument</td></tr>
<tr><td>Resources and training impact inserter confidence and ability</td><td>Increased support and resources for PIVC training is needed</td><td>Staff described deficits in PIVC training, mentorship, and resources (such as ultrasound) to support insertion success: ‘[I’m] not formally trained… on the job training.’ (C6). When the inserter was trained in ultrasound PIVC insertion, participants reported a preference for US insertion ‘it makes it easier’ (C2, C7). However, noted ‘it depends a little bit on the skill level and insight of the earlier operators’ training is adhoc with ‘little access to formal programs’(C7).</td></tr>
<tr><td></td><td>Many factors influence the successful implementation DIVA pathways</td><td>The current clinical environment and infrastructure does not support the longevity of a DIVA instrument ‘it might be used in research but once the study is complete will we be able to support its continued use?’. Existing policies and resources need to be reviewed as local change and implementation ‘is always going to be an issue’. Particularly after hours in the clinical setting ‘If it's after hours and there's no one else you might escalate things differently’(C4). Participants described needing team support and an appropriate environment.</td></tr>
<tr><td colspan="3">Key principles derived from above interview data
1. The final resource needs to support the longevity of its use and reuse in the clinical environment, leading to increased uptake and sustainability.
Recommendation: Create an instrument that considers available resources, is ‘future proofed’ and tailored to the hospital setting, and considers existing, related policies.
2. Across healthcare services increased educational training and resources need to be offered to both nurses and doctors to support improved PIVC insertion practices.
Recommendation: Improved training and educational resources, in addition to policy, will lead to better resource utilisation as well as an improved patient experience.</td></tr>
</table>
